# An evaluation of the feasibility of implementing a novel tobacco dependence treatment program for high-risk individuals into clinical practice within a community mental health center

**DOI:** 10.1186/s13033-022-00517-y

**Published:** 2022-02-20

**Authors:** Tory H. Hogan, Amanda Quisenberry, Nicholas Breitborde, Aubrey Moe, Amy Ferketich

**Affiliations:** 1 Division of Health Services Management and Policy, College of Public Health, The Ohio State University, 1841 Neil Ave, Columbus, OH 43085 USA; 2 Division of Population Sciences, Department of Health Behavior, Roswell Park Comprehensive Cancer Center, 665 Elm Street, Buffalo, NY 14203 USA; 3 Department of Psychiatry and Behavioral Health, College of Medicine, The Ohio State University, 1670 Upham Dr., Columbus, OH 43210 USA; 4 Division of Epidemiology, College of Public Health, The Ohio State University, 1841 Neil Ave, Columbus, OH 43210 USA

**Keywords:** Health care professionals, Community mental health centers, Mental health, Tobacco treatment, Tobacco cessation, Qualitative study, Psychotic-spectrum disorders, Schizophrenia, Implementation science, Feasibility

## Abstract

**Background:**

Individuals with serious mental illnesses experience deaths related to smoking at a higher prevalence than individuals without a psychotic-spectrum disorders. Traditional smoking cessation programs are often not effective among individuals with chronic mental disorders. Little is known about how to implement a tobacco cessation treatment program for this at-risk population within a community health center. The current study used qualitative methods to examine the factors that may enhance or impede the delivery of a novel tobacco cessation treatment for smokers with a psychotic-spectrum disorder diagnosis in an integrated care community health center.

**Methods:**

Using a case study design, we conducted 22 semi-structured interviews with primary care providers, mental health providers, addiction counselors, case managers, intake specialists, schedulers, pharmacists, and administrative staff employed at the organization. Interviews were transcribed and themes were identified through a rich coding process.

**Results:**

We identified environmental factors, organizational factors, provider factors and patient factors which describe the potential factors that may enhance or impede the implementation of a smoking cessation program at the integrated care community health center. Most notably, we identified that community mental health centers looking to implement a smoking cessation program for individuals with chronic mental health disorders should ensure the incentives for providers to participate align with the program’s objectives. Additionally, organizations should invest in educating providers to address stigma related to smoking cessation and nicotine use.

**Conclusions:**

The findings of our study provide valuable insight for administrators to consider when implementing a smoking cessation program in an integrated care community health center. Our findings provide public health practitioners with potential considerations that should be discussed when designing and implementing a smoking cessation program for individuals with chronic mental disorders.

**Supplementary Information:**

The online version contains supplementary material available at 10.1186/s13033-022-00517-y.

## Introduction

Compared to the general population, individuals with psychotic-spectrum disorders, such as schizophrenia and mood disorders with psychotic features, die approximately 15–20 years earlier than the general population [1], with an estimated 53% of these deaths due to smoking [2, 3]. About two-thirds of individuals with schizophrenia smoke, and less than 10% of ever-smokers with serious mental illness have quit, compared to almost 55% of ever-smokers in the general population [4–6]. As a result, this population is a priority population for effective tobacco cessation programs [7]. Community mental health centers are the ideal care site for tobacco cessation programs for individuals with psychotic-spectrum disorders, as they frequent these centers and there are providers in these settings who can appropriately monitor interventions and potential pharmacological side effects [8]. Patients with severe mental health illnesses who are seen at community mental health centers are less likely to be provided tobacco cessation programs, and these organizations struggle to implement tobacco cessation programs [9, 10]. When implementing tobacco cessation programs for the entire population served within community mental health centers, organizations face a multitude of barriers which include providers’ beliefs regarding patient’s lack of interest, belief that carrying out smoking cessation during patient visits will take up too much time, feeling there are too many demands already on staff to implement new programs, and a multitude of negative attitudes toward smoking cessation and whether or not it should be prioritized within the care setting [9, 11].

Deficits in cognitive functioning are the most frequently experienced mental health symptoms for individuals with psychotic-spectrum disorders, and the elevated rates of smoking in this population may stem [12], in part, from the positive effects of nicotine on cognitive function [13–15]. Traditional cessation programs do not address deficits in cognitive functioning—a factor that may contribute to the lack of effectiveness of these interventions among individuals with psychotic-spectrum disorders who smoke [16]. Outside of the smoking cessation literature, metacognitive remediation therapy (MCR)–an intervention providing clinician-delivered metacognitive skill development exercises that are mastered via practice using computerized exercises [16, 17] –has been shown to be effective in improving cognitive functioning for individuals with psychotic-spectrum disorders. Psychotic spectrum disorders include schizophrenia, schizophreniform disorder, schizoaffective disorder, or bipolar disorder with psychotic symptoms. [18, 19].

Drawing on these results, we developed a 12-week combination therapy that included traditional pharmacological treatment for smoking cessation (i.e., bupropion, varenicline, or nicotine replacement therapy) and a modified version of MCR [metacognitive remediation to quit (MCR-Q)] that is designed to promote development of skills and strategies to address specific real-world situations related to quitting smoking. In an open trial of MCR-Q, individuals with psychotic-spectrum disorders who smoke experienced reductions in nicotine dependency over the course of the intervention that were sustained six weeks after the completion of treatment [20].

For an effective smoking cessation program to have an impact, it must be easily incorporated into clinical practice. Barriers and facilitators to implementing tobacco dependence treatment in mental health and substance use treatment programs have been reported [21–25]; however, most of these investigations occurred in substance abuse treatment centers or are programs which do not address the unique tobacco cessation and health needs of individuals with psychotic-spectrum disorders. Little is known about the *feasibility* of implementing a tobacco dependence treatment program into integrated community health centers, which focus simultaneously on treatment of mental health, chemical dependency, and primary care, as well as provide employment and homelessness services. In addition, community health centers receive funding from multiple agencies and state and federal programming, making the management of such organizations complex. The little information that is available comes from facilities that specifically treat veterans [23, 25], who are a special population in their own right and have a unique service billing structure through the Department of Veterans Affairs.

Our public health system in the United States relies significantly on community health centers to provide primary care and mental health services to some of the most vulnerable populations in our society. More research exploring the implementation and feasibility of clinical care programs on our vulnerable populations will provide managers and administrators with guidance to facilitate the appropriate treatments for their clients. The purpose of this study was to explore the feasibility of implementing our combination tobacco cessation treatment for smokers with a psychotic disorder diagnosis in an integrated care community health center setting. Using a qualitative case study design approach, we interviewed employees in an integrated care community health center and identified potential barriers and facilitators to implementing the novel MCR-Q program into clinical care. The findings provide valuable insight for managers and administrators regarding the implementation of a smoking cessation program in an integrated care community health center.

## Methods

We used a case study design to explore the feasibility of implementing of our combination tobacco cessation treatment for smokers with a psychotic disorder diagnosis in an integrated care community health center setting. A case study design was chosen because it lends itself to understanding and capturing how interventions are implemented on the ground and explaining the complexities of a phenomenon in everyday contexts.

### Setting

The MCR-Q intervention was implemented at a community healthcare organization serving four counties in central Ohio. In Ohio, 20.5% of adults smoke, which is above the national average of 16.1, making smoking cessation an important public health initiative for community leaders and policy makers [26]. This particular organization was selected, in part, because it is a comprehensive provider of mental health, addiction treatment, primary care, dental services, pharmacy management, homelessness programs, and vocational training. The majority of clients are either experiencing homelessness or other forms of housing insecurity, and have major mental health and addiction needs. To meet the complex needs of its clients, the organization employs a wide variety of care providers, such as physicians, nurse practitioners, licensed social workers, case managers, psychiatrists, pharmacists, and clinical psychologists, who work inter-professionally to serve the needs of their community. Moreover, this organization provides a wide range of mental health, primary care, employment, and homelessness services to over 8000 adults who have a range of mental health disorders, including over 1000 with one of the psychotic-spectrum disorder diagnoses that we targeted in our smoking cessation program. Thus, it is truly an integrated physical and behavioral health setting built on the patient-centered medical home model [27]. The primary care services are provided at the organization’s Federally Qualified Health Center (FQHC).

The organization supports smoking cessation and felt strongly that their clients would benefit from evidence-based tobacco cessation programs that are effective among individuals who experience severe mental health disorders, which were prevalent among their client population. Prior to the study, primary care providers counseled smokers to quit and wrote prescriptions for cessation pharmacotherapy when appropriate. Additional services had been a struggle to adopt within the organization, as the current financial model made other counseling services and support groups unfeasible. Lastly, the organization had recently adopted a tobacco-free policy that included employees committing to be tobacco free. Employees who used tobacco were offered tobacco cessation programs through the organization’s insurance plan.

The foundation of the smoking cessation program is MCR, but it was tailored to promote quitting smoking (MCR-Q). Across the 12-session intervention, individuals participate in therapist-delivered metacognitive skills development exercises designed to facilitate improved knowledge and regulation of skills applied during completion of activities requiring the use of working memory, sustained attention, and behavioral disinhibition—three cognitive domains repeatedly linked to tobacco use [21]. Moreover, participants receive training in addressing intervening factors that may hinder a person’s ability to effectively apply these skills in real-life settings (i.e., emotion dysregulation, arousal dysregulation, and low motivation/self-efficacy). These skills are practiced in-session using computerized training exercises (Bracy, 2012) that require the use of these three cognitive skills to facilitate mastery. Conversations linking these skills to specific aspects of smoking cessation are included in each session to facilitate application of these skills to participants’ real-world smoking cessation activities. For example, utilizing a timed task that participants often report elicits increased autonomic arousal, participants have the opportunity to practice skills (e.g., diaphragmatic breathing) to cope with uncomfortable physiological sensations that may occur during smoking cessation attempts. Consistent with clinical guidelines for tobacco dependence treatment [22], these psychotherapeutic activities are complimented with concurrent participation in smoking cessation pharmacotherapy in the form of bupropion, varenicline, or nicotine replacement therapy.

### Interview procedures

The current interview sub-study was approved by the Institutional Review Board of the Ohio Department of Health. Interviews were conducted by one investigator THH using a face-to-face format at the organization’s office where the cessation program was offered. To minimize the burden on participants, interviews lasted approximately 30 min and participants received a $20 gift card. We developed a semi-structured interview guide (provided in Additional file 1) that assessed perceptions of barriers and facilitators of implementing the program within the organization. Interviews were digitally recorded and then transcribed by three undergraduate students.

### Participants and recruitment

Using purposeful sampling [28], we interviewed primary care providers, mental health providers, addiction counselors, case managers, intake specialists, schedulers, pharmacists, and administrative staff at the organization. We selected this sampling approach because of its ability to identify participants who share the same experience of a central phenomenon of interest [28]. This enabled us to identify participants who would contribute to a shared understanding of the organizational context for implementing a tobacco cessation program for individuals.

Potential interviewees were identified in two ways: First, the organization’s leadership, along with the intervention therapy team leadership, developed a list of roles/titles in the organization that would be able to speak to a wide array of topics regarding the implementation of a novel tobacco cessation program. The research team wrote a recruitment email, which explained the goals and objectives of the study to the organization’s leadership team. The leadership team sent a recruitment email to all potential employees using their internal e-mail system. Individuals responded to the email and directly scheduled their interview with the study interviewer THH. Based on input from the intervention leadership, the lead qualitative evaluator THH and the organization leadership team, the following individuals were purposefully recruited: individuals who worked in roles within the organization related to tobacco cessation (e.g., nurses, physicians, advanced practice providers), worked with clients who may utilize tobacco cessation services (e.g., case managers, counselors), or were in administrative positions that could provide insight into the organization’s ability to implement the program and manage the patient processes (e.g.., scheduling). Additionally, two weeks after the initial recruitment email was sent, the lead qualitative investigator followed up the intervention therapy leadership team and showed the team a list of titles of participants which had participated or were scheduled to participate. Together, the group brainstormed additional individuals from the organization who had not yet responded to the first recruitment e-mail, and we sent an additional recruitment email directly to this list of individuals. This ensured we had adequately sampled participants who could address the complexity related to the clinical and administrative needs related to implementing the MCR-Q program in the organization. Questions related to the program were asked, including resources available, barriers, financial impact, training of staff, and staff attitudes towards a new tobacco cessation program.

We conducted interviews prior to the start and at the end of the MCR-Q program. A total of 22 interviews were conducted and were deemed appropriate because the study had reached saturation [29]. The final interview sample included three case managers, two nurse practitioners, two therapists, two team leaders, two residential specialists, two cognitive enhancement therapy workers, one pharmacist, one primary care physician, one residential program manager, one intake coordinator, one receptionist, one assistant clinical director, one clerk, one recovery guide, and one social worker. Considering the pharmacological intervention component of the MCR-Q intervention, and the importance of gaining the clinical perspective, we specifically note that a majority of the clinical team who would be responsible for providing medication management within the primary care setting of the organization participated in the study. Our interview guide did not ask participants if they were smokers, but during the semi-structured interviews, 14 participants voluntarily shared that they did not “smoke cigarettes” or “smoke”, and two reported that they had recently quit smoking. We anticipated further sampling after our initial coding; however, the interviews were consistent and similar in themes, and we were hearing the same comments among participants. Therefore, we believe we had met saturation [30]. This study was approved by the Ohio Department of Health Institutional Review Board.

Prior to analyzing the data, a team of three students transcribed the interviews. After each student transcribed an interview, a different student reviewed the transcript and audio file for inaccuracies using a protocol developed by the first author.

As suggested by Creswell [29], the analysis proceeded through the following steps: (1) preliminary examination of the data where the primary author reviewed the transcribed interviews and took notes; (2) coding each interview within each case; (3) using the codes to develop themes and sub-themes; (4) verifying the themes and sub-themes with other members of the research team; (5) connecting and inter-relating themes; and (6) constructing a case study table consisting of themes, sub-themes, and illustrative quotes for each case. Our results are reported in a rich description of each theme, explaining examples as well as supporting each theme with illustrative quotes which are outlined in the Table 1. The data verification process included confirming the interviewer’s understanding of the interviewee’s response by summarizing and paraphrasing throughout the interview [31], crafting rich descriptions of the interviews, and confirming information from the interviews with MCR-Q team members.

**Table 1:** Themes and sub-themes

Theme	Description	Example quotation
*Reimbursement Mechanism*	The organization’s ability to implement the program will be based on whether they can receive reimbursement for MCR-Q	“It’s difficult because [we’re an] FQHC so um you know HRSA really decides what we do with our money.”
**Organizational issues**		
*Multi-disciplinary provider teams*	The organization has multi-disciplinary teams (FQHC & Mental Health and Addiction Services), which is conducive to a smoking cessation program which requires a combination of medication and therapy	“Okay. Well we already have a lot of integration between the two sides. You know they were behavioral health for a long time and then they added primary care later. Um, so probably things that we have going first would be a good place to do it, cause we have like a whole integrated team that we have, approach with our patients.”
*Aligning the human resource incentive program with MCR-Q*	The organization utilizes productivity hours to measure staff productivity throughout with the exception of the primary care clinic and the program would need to align with this incentive structure	“I think XXXXX [Organization name] would really have to say hey this is what we’re gonna do for you if you do this. We’re gonna give you this much productivity for it. A lot of it depends on the productivity that’s gonna come out of this for the people.”
**Provider behaviors**		
*Lack of time*	Team members expressed a lack of time or resources to making smoking cessation a priority	“I think you know, um, the challenge would be the case workers saying, this is one more thing I have to do. That would be a big one.”
*Buy In/attitudes of providers and/or team members*	Various team members have different levels of willingness to incorporate the program, and varying attitudes toward adopting this new program	“Yeah. But with the therapists you know I think most enjoy learning and trying out new things so.”
*Provider prescribing behaviors*	Getting providers who prescribe to change behaviors to incorporate potential side effects/interactions with smoking cessation drugs	“Um but help them understand that when they’re prescribing if they have a client in the smoking cessation program under Bubenorphine to please be willing to adjust the psychiatric meds as best able to support the program.”
**Client concerns**		
*Motivation to quit smoking*	Getting clients to be motivated to have an intention to quit	“So, I think it’s—it’ll be difficult to try to get them to understand that they really need, you know, to stop smoking.”
*Competing client social needs and priorities*	Due to complex social needs (housing, transportation) of the clients, smoking cessation may not be feasible or the most appropriate need to address during clinic interactions	“I’ve always said it’s hard to stop drinking when you’re sleeping under a bridge it’s probably going to be hard to stop smoking too.” “Just our population can be difficult and you know just being mindful of that. Uh they have a lot of barriers you know whatever their trying to do. Um you know a lot of our people are homeless who come here.”

## Results

Through our analyses, we identified 4 themes: *reimbursement mechanisms, organizational issues*, *provider behaviors,* and *client concerns*. Within these, we identified 8 sub-themes. Table 1 contains the themes and any sub-themes that emerged from the interviews, along with illustrative quotes. In the next few sections, we describe our assessment of the issues that are important to consider and understand when implementing the MCR-Q program into clinical practice.

### Theme 1: reimbursement mechanisms

This theme describes issues related to being able to receive reimbursement for components of the MCR-Q program. Participants stated that the organization’s ability to implement a program was based on whether they could receive reimbursement for MCR-Q, and the specific components involved. This was described by participants as both a potential facilitator or barrier to implementing the MCR-Q program. As an FQHC, the organization relies primarily on Medicaid and Health Resources and Services Administration (HRSA) funding mechanisms which have specific billing requirements. Activities which can be billed for are easier to adopt into practice in the organization. Activities which are not able to be billed for are a struggle to adopt into practice in the organization. At the time of the interviews, the research team was able to confirm that the activities of the program would meet the requirements of the tobacco cessation programs for these reimbursement mechanisms. HRSA provides funding for the primary care center within the comprehensive care clinic. Medicaid reimburses for the addiction and behavioral health services offered at the comprehensive care clinic. When evaluating the feasibility of the MCR-Q program, the ability to be reimbursed is a significant consideration that participants described.

### Theme 2: organizational issues

This theme describes issues related to how the organization operates that are important to consider when evaluating the feasibility of the MCR-Q program. Two sub-themes, *aligning the human resource incentive programs with MCR-Q* and *multi-disciplinary provider teams* are included in this theme.

*Sub-Theme 1: aligning the human resource incentive programs with MCR-Q* describes the importance of the MCR-Q program being included in the organization’s employee work incentive program. Participants explained that the organization utilizes productivity hours as the basis for salary and compensation for many employees. Employees must track productivity hours for services they provide to clients. For example, when a therapist spends one hour leading a group therapy session, they receive one productivity hour. Each staff member has a goal for monthly productivity hours, and upon meeting each monthly goal, they receive additional compensation. Employees would need to be able to receive productivity hours for their efforts related to the MCR-Q therapy program for the program to be successful at their organization. Some participants stated that if employees could not receive productivity hours for the MCR-Q program, then the program would most likely not be successful.

*Sub-Theme 2, multi-disciplinary provider teams* describes how the organization has multi-disciplinary teams which are comprised of primary care, mental health, and addiction services providers which is an amenable structure for the MCR-Q program and the multi-disciplinary clinicians needed to implement such a program. There was a consensus among participants that the new program would not require any new hiring within the organization. Additionally, within this theme, some participants noted that some of the teams had prior experience offering tobacco cessation programs which may have incorporated some of the techniques of the program.

### Theme 3: provider behaviors

This theme describes issues regarding provider behavior, and how that behavior may impact the implementation of a new tobacco cessation program. Providers refer to anyone in the organization who offer a service to a client, such as social workers, primary care providers (MD, DO or Nurse Practitioner), pharmacists, psychiatrists, case managers, and counselors. There are three sub-themes in this theme: *buy-in/attitudes of providers and team members, provider prescribing behaviors,* and *lack of time*.

*Sub-theme 1: buy-in/attitudes of providers and team members* describes the role of the providers’ and other team members’ personal perceptions and attitudes toward tobacco cessation and the importance of providing tobacco cessation during a clinic visit. The study site used inter-professional teams comprised of social workers, primary care providers (MDs and NPs), pharmacists, psychiatrists, case managers, and counselors, and each team member needs to be aligned with the broader patient care goals and care needs. The new MCR-Q program has the potential to impact how some of these individuals provide care; for example, the psychiatrist may need to adjust their current clinical treatment plans or be aware that a client could be more irritable while trying to quit smoking and adjust expectations accordingly. This may subsequently require more time from a provider or a team member and make the patient more demanding during one-on-one treatment sessions or other services provided by someone on the interprofessional team. During our interviews, participants made note that the program would need to address this issue if it were to be successful. For example, team members suggested that despite their strong relationship with their clients, this intervention would require the cooperation of multiple team members and the belief from all team members that tobacco cessation was important.

*Sub-theme 2: provider prescribing behaviors* describes how the new tobacco cessation program would require providers who are responsible for managing the pharmacological components of a client’s treatment to potentially be open to changing the medications they prescribe. Participants indicated that the medications needed as part of the MCR-Q (bupropion, varenicline, or nicotine replacement therapy) program may interact with the current prescriptions used to manage the patients’ psychotic-spectrum disorders. Providers, specifically those with a role in medication management, may have to make these changes if an individual’s current medications have interactions with the medications included in the MCQ-R program. Participants described how a vital part of the program being successful will fall on the willingness and ability of prescribing providers to adjust medications, if necessary, due to a person quitting smoking, taking cessation pharmacotherapy, or experiencing an adverse event to a cessation medication [32]. There was a belief among participants (among both clinicians who prescribe and those who do not have prescribing responsibilities) that providers in their organization get into routines regarding the medications they prefer to prescribe or have certain medications that they prefer over others. This is often based on their personal experience with prescriptions or, as one participant stated, “because we like to use the things we know.”

*Sub-theme 3: lack of time* describes how participants stated that a barrier to successful implementation of the tobacco cessation program was a lack of time during their day to devote to adopting the program and incorporating it into their interactions with clients. Participants stated that this would be “one more thing” for them to do, within a long list of job tasks. Participants described being open to the treatment, since quitting smoking was an important issue for their clients, but also expressed difficulty in understanding how they would be able to continue to do what they are already doing while adding another task to their list of responsibilities.

### Theme 4: client concerns

Client themes describe perceived issues related to the client population that would impact the implementation of a new tobacco cessation program. Two sub-themes emerged as a part of this theme: *competing client needs and priorities*, and *motivation to quit smoking*.

*Sub-theme 1: competing client needs and priorities* describes how there are competing health and social needs of individuals with psychotic-spectrum disorders who may be seeking tobacco cessation treatment. Study participants expressed that due to the complex health and social needs of their clients, it may not be feasible to address tobacco cessation during client interactions. Many of the clients who receive care at this organization deal with homelessness, food insecurity, legal issues, or have other issues, which they consider higher priority. Additionally, the clients targeted in the MCR-Q intervention often have a co-diagnosis that creates complex health needs. Two participants referenced the “Maslow’s hierarchy of needs” with regard to the program and suggested that tobacco cessation may be lower on the hierarchy. For example, a provider explained that when a client comes to a visit and shares that they have recently lost their home and during that process they lost their medications, that visit is spent helping the client find a place to live and helping them get re-fills on their medications. These needs make it difficult to prioritize tobacco cessation activities.

*Sub- theme 2: motivation to quit smoking* describes how clients targeted for the MCR-Q treatment may not be interested in quitting smoking and this may make it difficult to recruit individuals to the treatment program. Participants explained that the organization would need to encourage and support clients and educate them about the program to improve the motivation to quit using tobacco products among this client population. Some reasons clients may not be interested include that many enjoy smoking or relate tobacco use to positive symptom management outcomes, which specifically should be addressed when considering how to get clients to consider the MCR-Q program.

## Discussion

This study is responsive to critical gaps in the implementation of tobacco cessation for people with psychotic-spectrum disorders—the need for an effective tobacco cessation program, and the need for evidence to support the implementation of such programs within community health centers. Translating what is learned from research into real world settings is far more complex and challenging than it appears [33]. Integrated community health centers face limited financial and human resources, making it difficult to have the bandwidth needed to change clinical care practices and implement new care practices [34]. Our findings identify key factors to consider when planning to adopt the MCR-Q program in an integrated community health center. We identified four themes: *reimbursement mechanisms, organizational issues*, *provider behaviors,* and *client concerns*, which provide insight for leaders planning to implement the MCR-Q program. Our findings are consistent with implementation science studies which have found that patients, providers, organizations, and the payer environment play a vital role when implementing a new evidence-based practice into care [35].

Our study found that staff perceive reimbursement mechanisms as being an important factor to consider when evaluating the feasibility of implementing an MCR-Q program. Federal and state policy makers determine HRSA and state Medicaid reimbursement policies, which are responsible for determining the services within the MCR-Q program for which community mental health centers could bill. It is common for community mental health centers to rely on two major revenue sources: HRSA reimbursement for the organization’s FQHC activities and Medicaid for mental health and substance abuse services. The MCQ-R program would require a primary care clinician, psychiatrist, or mental health nurse practitioner to prescribe cessation pharmacotherapy, provide medication management, and troubleshoot with clients who may need medication adjustments as a result of either quitting smoking or side effects of the cessation pharmacotherapy. This finding suggests that integrated community health clinics have a strong need for any tobacco cessation programs to work within the already existing reimbursement mechanisms. This would be a key matter for all organizations considering the MCQ-R program.

Next, we identified two themes which we considered to describe organizational issues that should be evaluated as part of understanding the feasibility of the MCR-Q- program. The bulk of tobacco cessation programs are implemented at primary care offices, substance abuse treatment centers, and mental health clinics. Our study was the first to examine the feasibility of implementing a combination tobacco cessation therapy in a comprehensive community care clinic which offers integrated mental health, substance abuse treatment, and primary care under a single organizational entity. We found that staff at our organization believed that their organization’s multidisciplinary approach to patient care was an added benefit and made their organization a good fit for the MCR-Q program, which incorporates pharmacological with behavioral health interventions into a single treatment.

We also found that participants perceive a struggle to get buy-in from providers, while other employees perceive providers to be very supportive. These findings are consistent with previous studies which have identified a multitude of barriers to the provision of tobacco cessation programs among clinicians working with underserved communities [30]. During our interviews, it was suggested that if the community mental health center was going to implement such a program, there needed to be a focus on gaining buy-in and improving the attitude of providers. These findings are in line with research examining clinician perceptions and implementation barriers for tobacco cessation programs in community mental health centers, which has cited discourse suggesting that smoking may help individuals with serious psychotic disorders as it may have a therapeutic component, or quitting smoking may be dangerous for clients as it could put them under stress [11, 36, 37]. When considering implementing the MCQ-R program, it is critical for administrators to understand this barrier, as there is not definitive consensus on treatment initiation within this client population, which may impede the role these providers are willing to play in recommending this treatment or initiating the care. Additional education, or further understanding providers’ specific concerns, could help the organization design educational content and training opportunities to manage this barrier. Further studies looking to implement an MCQ-R program should strongly consider establishing a training mechanism for individuals within the organization to ensure that all individuals along the care continuum are able to understand the benefits of the MCR-Q treatment. This could also ensure that clients are appropriately referred to the program.

Our study identified that providers may struggle to implement a tobacco cessation program due to the *competing client needs and priorities* they must manage. This finding is consistent with previous research which has identified that providers in community mental health centers believe tobacco cessation services are not a priority for their clients [11]. Yet, previous research found that few mental health providers ask clients about smoking and tobacco cessation readiness [31]. When considering our finding related to the client needs and the clinician interpretation of their priorities, further research could explore whether or not patient attitudes are impacted by the fact that providers aren’t likely to ask clients about smoking and tobacco cessation. Tobacco cessation often takes lots of repeated attempts and engaging with clients multiple times before they decide to partake in cessation programs. Given the target population for the MCR-Q therapy and the unique needs of these individuals, community mental health centers need to think broadly about how they can incentivize their providers to value tobacco cessation assessment (e.g., exploring the topic and interest with clients) and engage with clients about discussions that could lead to the participation in cessation programs. Additionally, when considering our other findings regarding the challenges providers face (lack of time), prioritizing tobacco cessation is a challenge in these organizations. Part of the considerations management should consider is how they can potentially alleviate the resource (time) constraint of clinicians so tobacco cessation services can be provided. Finding ways to align the provider with client goals for tobacco cessation and shift the perceptions of providers within community mental health centers must be a consideration when implementing a tobacco cessation program. It is unclear if our findings are a result of a lack of client readiness or of the personal attitudes of the individuals who participated in our study. It is also not clear if more resources for the provider would mediate this issue and potentially enable the provider to have more time with the patient which could be used to address tobacco cessation.

Our findings provide valuable insight into the implementation of new clinical programs in community mental health centers. The successful implementation of evidence-based practices in community mental health settings is based on a multitude of implementation domains, such as leadership, workforce, workflow, and reinforcement, and the ratio of barriers and facilitators within an organization [35]. Organizational capabilities, including the time providers have to meet the care needs of clients, the human resource management systems in place to incentivize employee performance, and the types of providers employed, are needed to implement programs for clients with complex mental health needs, such as psychotic-spectrum disorders. Additionally, community mental health centers which do not have large, inter-disciplinary teams or the ability to work closely with a client’s primary care clinician may not be able to successfully implement the MCR-Q program, or other tobacco cessation programs which include a pharmacology component. Strategizing to overcome these potential barriers should be a key priority for individuals leading the implementation of a tobacco cessation program in community mental health centers.

### Limitations

Our first limitation is that we only conducted interviews with staff at one study site. This meant that there was a limited sample of individuals who met the criteria to be included in our purposeful sampling approach. Given the limited potential pool of participants, this may have influenced the amount of variability in our participants’ responses. In addition, participants were all employed by the community mental health clinic, which may bias their responses to the interview questions. However, they were assured that their responses were anonymous, that all results would be reported in aggregate, that the organization’s leadership would not know they had participated in the study, and that participation would not affect their employment. Lastly, we did not systematically collect information on participants’ smoking status or tobacco use history, though some participants voluntarily shared this as part of their responses. We acknowledge that individuals’ tobacco use may have impacted their responses to questions regarding the implementation of a tobacco cessation program, and we do not know the extent to which results are indicative of personal factors related to tobacco use.

## Conclusion

Overall, this study provides insight into factors that community mental health administrators and leaders should consider when planning to implement a tobacco cessation treatment targeted at clients with serious mental health disorders. When considering our findings, leaders of community health centers and mental health clinics may want to ensure that, in addition to the tobacco cessation services, the organization engages providers in education and training regarding their attitudes toward tobacco cessation, meets regularly with interdisciplinary team leaders to ensure all elements of the organization’s incentive structures are aligned to support success of the program, and ensures that the organization is supporting team members’ commitment to such a service.

## Supplementary Information

**Additional file 1.** An evaluation of the feasibility of implementing a novel tobacco dependence treatment program for high-risk individuals into clinical practice within a community mental health center.

## Data Availability

The data analyzed during the current study are available from the corresponding authors on reasonable request.

## Abbreviations

MCR: Metacognitive remediation therapy
MCR-Q: Metacognitive remediation therapy to quit
