# Supplementary material for: An evaluation of the feasibility of implementing a novel tobacco dependence treatment program for high-risk individuals into clinical practice within a community mental health center
Source: Int J Ment Health Syst. 2022 Feb 20;16:15. doi: 10.1186/s13033-022-00517-y (PMC8858522; doi:10.1186/s13033-022-00517-y)
Supplement: Supplementary file 1 — Additional file 1. An evaluation of the feasibility of implementing a novel tobacco dependence treatment program for high-risk individuals into clinical practice within a community mental health center. [file 13033_2022_517_MOESM1_ESM.docx]

An evaluation of the feasibility of implementing a novel tobacco dependence treatment program for high-risk individuals into clinical practice within a community mental health center

Tory H. Hogan

[Hogan.323@osu.edu](mailto:Hogan.323@osu.edu)

Amanda Quisenberry

[Amanda.Quisenberry@RoswellPark.org](mailto:Amanda.Quisenberry@RoswellPark.org)

Nicholas Breitborde

[Nicholas.breitborde@osumc.edu](mailto:Nicholas.breitborde@osumc.edu)

Aubrey Moe

[Aubrey.moe@osumc.edu](mailto:Aubrey.moe@osumc.edu)

Amy Ferketich

Ferketich.1@osu.edu

Qualitative Interview Script ID: ____________ Date: ______________ Approval date: 5/24/2017

Good **[morning, afternoon, evening] [Provider’s name]**.

My name is **[facilitator’s name]** and I will be conducting the interview today. I would like to thank you for taking the time to do this interview. During this interview, we will talk about your experience working in a clinical setting and incorporating a new program into clinical practice.

I will ask you a series of questions and you can choose to respond as you wish. All of your responses will remain confidential. If you wish to end the interview at any time, please let me know.

The interview will last up to 30 minutes. We are audiotaping the interview so that afterwards a transcript of the discussion can be created. In the analysis, no individual will be identified and all information will be reported in aggregate.

May we begin?

**Interview Questions**

Before I start asking you questions, I would like to summarize the new smoking cessation program that will start this spring at Southeast. This program is designed for smokers who have a diagnosis of schizophrenia, schizoaffective disorder, or bipolar disorder. Because standard programs that include medication and cognitive behavioral therapy have not historically helped smokers with a serious mental illness quit, new programs are needed. The program that my colleagues at Ohio State will introduce at Southeast includes a standard smoking cessation medication, bupropion, and a new form of counseling called metacognitive remediation therapy. This treatment has been developed as a treatment for individuals with schizophrenia and it has also been found to be effective for treating bipolar disorder. It essentially involves administering a series of computer games that train individuals on memory. The counselor, who is present during the session, guides the individual through problem solving and translation of skills acquired to other areas of life. The program has been found to improve memory and attention and for these reasons, we think it may help smokers quit.

I would now like to discuss how such a program can be incorporated into the clinic setting.

1. What role do you play at Southeast in the care of clients?

2. What resources are available at Southeast to implement this type of program?

3. Is there anything that Southeast would have to give up in order to adopt this program?

4. If so, what are the trade-offs?

5. What will this program mean from a financial standpoint?

6. Do you think there can be reimbursement for this type of program

7. What is the process for introducing a new program into your billing system?

8. How are staff typically trained on new types of therapies?

9. How does this therapy compare to others that are used in practice?

10. Do you think the counselors at Southeast will be excited or resistant to this program?

11. Given our interest in implementing the new therapy at Southeast, is there anything else that you think we should know?
